# Mystics and politics: women and the interpretation of the Scriptures

**DOI:** 10.1515/sem-2025-0093

**Published:** 2025-05-30

**Authors:** Jenny Ponzo

**Affiliations:** Università degli Studi di Torino, Torino, Italy

**Keywords:** mysticism, interpretative style, enunciation, authority, author, mysticisme, style interprétatif, énonciation, autorité, auteur

## Abstract

In cultures based on foundational or sacred texts, the contrast between different interpretative styles is the crux of important political questions: who has the right to interpret the sacred texts, and therefore to exert authority over the community? Which Senders have the right to sanction interpretations? In Roman Catholic culture, two main interpretative styles can be identified. The first is the intellectual and rational approach that most characterizes theological discourse, the Magisterium and canon law; the other is the experiential and mysterious approach characterizing mystical discourse, which is scrupulously – and sometimes severely – evaluated by the ecclesiastical authorities, especially in the case of women interpreters. In this paper, I look into one facet of the clash between these two interpretative styles. I first present some reflections about the relationship between holy characters and political engagement, pointing out how and why mystics are endowed with a particular authority and leadership in their communities; I then focus on the mystical interpretative style and related enunciative strategies, showing how the authority of the woman interpreter is built through a stratification of the authorial subjectivity, with particular reference to the case of Maria Valtorta.

## Résumé

Dans les cultures basées sur des textes fondateurs ou sacrés, le contraste entre les différents styles interprétatifs est le nœud d’importantes questions politiques: qui a le droit d’interpréter les textes sacrés, et donc d’exercer une autorité sur la communauté ? Quels Destinateurs ont le droit de sanctionner les interprétations ? Dans la culture catholique romaine, deux styles interprétatifs principaux peuvent être identifiés. Le premier est l’approche intellectuelle et rationnelle qui caractérise le plus le discours théologique, le Magistère et le droit canonique; l’autre est l’approche expérientielle et mystérieuse qui caractérise le discours mystique, lequel est scrupuleusement – et parfois sévèrement – évalué par les autorités ecclésiastiques, en particulier dans le cas des femmes interprètes. Dans cet article, j’examine une facette du conflit entre ces deux styles interprétatifs. Je présente d’abord quelques réflexions sur la relation entre les saints et l’engagement politique, en soulignant comment et pourquoi les mystiques sont dotés d’une autorité et d’un leadership particuliers dans leurs communautés. Je me concentre ensuite sur le style interprétatif mystique et les stratégies énonciatives associées, en montrant comment l’autorité de la femme interprète se construit à travers une stratification de la subjectivité, en particulier dans le cas de Maria Valtorta.

## 1 Introduction

In cultures revolving around one or more foundational or sacred texts, it is possible to identify multiple interpretative styles which are in competition with each other.1The research presented herein was carried out in the framework of the project NeMoSanctI, which has received funding from the European Research Council (ERC) under the European Union’s Horizon 2020 research and innovation programme (grant agreement No 757314). Such cultures can be imagined as semiospheres (Lotman 2000 [1992]) with a simple structure: the sacred or foundational texts occupy a central position and different metatexts (Genette 1982) and communities of interpreters are placed in more or less peripheral positions around them. Each group applies different criteria, assumptions, and approaches to the foundational texts that give rise to different readings, with important cultural, social and political consequences. Indeed, interpretative styles not only determine readers’ relationship with the fundamental text, they also constitute an important basis for defining group identity and can influence the relationship among the interpreters and, consequently, the organization of society into distinct and hierarchically organized classes or categories.

Significant reflection on such interpretative issues concerning foundational texts can be ascribed to anthropologist Vincent Crapanzano (2000), who focused on literalism in America. Considering literalism an “interpretative style,” Crapanzano studied it in relation to both the Bible and the Constitution. Other studies in the field of the semiotics of religion have explored similar interpretative dynamics. For instance, Ponzo (2019) tackles a sample of contemporary apologetic texts and shows how the same biblical verses about the phenomenon of tongues are interpreted very differently by Conservative Evangelicals and Charismatics. The former are bound to an interpretation of the sacred text based on the criteria of plain and literal meaning, while the latter seek to directly experience the spiritual phenomena described in the New Testament and particularly Acts, with the result that interpretation is considered successful if it leads to a “felicitous” (Austin 1962) spiritual performance. Therefore, the very same verses are used to reinforce opposing arguments: one community of interpreters has posited them as the basis for rejecting and condemning glossolalia, while the other has used them to justify and promote the same practice. The application of different interpretative styles is linked to a radically different vision of religious life, but the enunciation of the interpretative criteria can also be a constitutive part of the argumentative apparatus. In some cases, as in fundamentalist discourses,2For a semiotic approach to fundamentalist discourse, see Massimo Leone’s works, in particular Leone (2012). the interpretation of small portions of the sacred text ends up being more of a rhetorical device for asserting a certain thesis that seeks legitimacy in the sacred text, interpreted *in a certain way*, than the coherent application of a specific methodology of interpretation of the text itself. As these extreme cases clearly show, the contrast between different interpretative styles is the crux of important questions of an exquisitely political nature: who has the right to interpret the sacred texts, and therefore to exert authority over the community? What are the parameters that guarantee the legitimacy of the interpretation? Which “Senders” have the right to “sanction”3This is a reference to the narratological terminology proposed by Greimas (1983). interpretations?

Umberto Eco (1990) identified two main interpretative trends in western culture; one is rational and based on the *modus ponens*, whereas the other is hermetic, related to secrecy, mystery, and polysemy. Similarly, and more specifically, two main interpretative styles can be identified in Roman Catholic culture. The first is the intellectual and rational approach that most characterizes theological discourse but also the Magisterium (i.e., the Pope’s official and authoritative discourse) and canon law, and the other is the experiential and mysterious approach that characterizes mystical discourse in a special way. Even though the foundational text is constituted by the Bible, the Church does admit the possibility that forms of individual revelation may still take place; however, it maintains that individual cases must be scrupulously examined on the basis of rational and even judicial criteria, such as authenticity and consistency with doctrine.4The Catechism of the Catholic Church (III, 67), for instance, states that:

Throughout the ages, there have been so-called ‘private’ revelations, some of which have been recognized by the authority of the Church. They do not belong, however, to the deposit of faith. It is not their role to improve or complete Christ’s definitive Revelation, but to help live more fully by it in a certain period of history. Guided by the Magisterium of the Church, the sensus fidelium knows how to discern and welcome in these revelations whatever constitutes an authentic call of Christ or his saints to the Church. Christian faith cannot accept ‘revelations’ that claim to surpass or correct the Revelation of which Christ is the fulfilment, as is the case in certain nonChristian religions and also in certain recent sects which base themselves on such ‘revelations’ (https://www.vatican.va/archive/ENG0015/__PH.HTM).

The issue of the legitimation of the authority that can establish the right way of interpreting the Scriptures is addressed in Eco’s essay about the symbolic mode (Eco 1984). In particular, focusing on the Medieval Christian culture, Eco points out that the Church based its interpretative authority on the Tradition,

but the Tradition was represented exactly by the series of the ‘good’ interpretation of the Holy Scriptures. In other words, the Tradition draws its right to control the interpretation of the Books from the interpretation of the Books. Quis custodiet custodes? How can the authority legitimate the interpretation, since the authority itself is legitimated by the interpretation? … The only possible answer to this question was a practical one: the rules for good interpretation were provided by the gatekeepers of the orthodoxy, and the gatekeepers of the orthodoxy were the winners (in terms of political and cultural power) of the struggle to impose their own interpretation. (​Eco 1984​: 150–151)

In the hagiographies of mystics in all historical periods, the severe judgment and sometimes open hostility of the Church’s authorities is a recurrent topos. When the mystics in question are women, gender biases also influence the role of the interpreter and the Sanction of the ecclesiastical authority.5For further reflections on the Church’s Sanction of saintly figures proposed as exemplary interpreters of the Scripture, on the controversial role of women interpreters, and the issue of women’s predication (with a focus on Domenica da Paradiso), cf. Ponzo (forthcoming).

In this article, I look into a specific facet of the clash between these two interpretative styles in Catholic culture. I first present some reflections about the relationship between Catholic holy characters and political engagement, pointing out how and why mystics are endowed with a particular authority and leadership in their communities; I then focus on the mystical interpretative style and related enunciative strategies, showing how the authority of the interpreter is built through a stratification of the authorial subjectivity. Finally, I devote some attention to the Sanction of this kind of interpretation by the Church, with particular reference to the case represented by Maria Valtorta.

## 2 Maria Valtorta: a controversial author

Maria Valtorta (1897–1961) was an Italian lay mystic and a prolific writer.6A digital version of Maria Valtorta’s works is available at the following websites: https://scrittivaltorta.altervista.org/index.htm; http://www.valtortamaria.com/. She is mostly known for being the author of a rewriting of the Gospels (*L’Evangelo come mi è stato rivelato*, first published in four volumes between 1956 and 1959) that she presented as the fruit of revelation. She also wrote exegetic works, such as *Lessons about Paul’s Epistle to the Romans* (Valtorta 2008, first published in 1977), and *The Book of Azariah* (Valtorta 2007, first published in 1982) in which she comments on 58 masses. This latter text is the focus of my analysis here. For many years, Maria Valtorta sought to obtain the Church’s official approval for her writings. During a private audience in 1948, Pope Pius XII said that Valtorta’s *Evangelo* could be published as is, without comments on its origins, and that readers would understand it. He did not suggest erasing the references to visions and other forms of divine communication received by Maria Valtorta in the narration, but he stated that the preface should not include an explicit reference to the book’s supernatural inspiration. The following year, however, the Holy Office prohibited the publication of *Evangelo* and, after its unauthorized publication, put it on the Index of prohibited books. An anonymous January 6, 1960 article in the *Osservatore Romano* (the official newspaper of the Holy See)7https://clubamicivaltortiani.forumfree.it/?t=61729539. commented on the prohibition, pointing out that this ban was appropriate given the excessive length and prolixity of the discourses attributed to Mary and Jesus, inconsistent with their humility and modesty, the “almost gossipy” tone of the narration, the scandalous and non-edifying nature of some episodes, and the inaccuracy of certain historical and geographical details. The final part of the article also stresses as problematic the identity of the writer and her claimed relationship with the sacred characters, together with her disobedience and irreverence in relation to the ecclesiastical authority:

The Work, therefore, would have merited condemnation even if it were only a novel, if for no other reason than irreverence. In reality, however, the author’s intentions go even further. Scrolling through the volumes, here and there we read the words “Jesus says…,” “Mary says…”; or “I see…” and the like. Indeed, toward the end of volume IV (p. 839), the author reveals herself to be… a woman and writes that she witnessed the totality of Messianic time and that her name is Maria. These words bring to mind the fact that, approximately ten years ago, certain voluminous typescripts were circulating that contained purported visions and revelations. It transpires that, at the time, the pertinent Ecclesiastical Authority forbade the printing of these typescripts and ordered that they be withdrawn from circulation. Now we see them reproduced in almost their entirety in the present Work. Therefore, this public condemnation by the Supreme Holy Congregation is all the more fitting because of this grave disobedience. (my translation)^7^

The Index was abolished in 1966 but, even though it lost its legal value, the Church has underlined its enduring moral value. In 1985, Joseph Ratzinger, then-Cardinal Prefect of the Congregation of the Holy Doctrine, claimed in an official letter8http://www.internetsv.info/Valtorta.html. that, even though the Index had been abolished, the diffusion of Maria Valtorta’s work was not seemly because it could be harmful to the less well-educated and informed members of the faithful. In 1992, Cardinal Dionigi Tettamanzi, General Secretary of the Italian Episcopal Conference, wrote a letter to the editor of Maria Valtorta’s work asking that

precisely for the true good of readers and in the spirit of genuine service to the faith of the Church, I would request that in any reprinting of the volumes, it should be clearly stated at the outset that the “visions” and “dictations” referenced in them cannot be considered to be of supernatural origin, and must instead be regarded as nothing more than literary forms the author has used to narrate, in her own way, the life of Jesus.

This position seems to be quite similar to the one first expressed by Pius XII: the Church was potentially willing to approve the publication of Valtorta’s work provided that no claims about its origin be asserted in the preface. In other words, the Church was prepared to allow the publication of the work itself on the condition it not include any paratext containing a Sanction that contradicted the Church’s own Sanction and thus challenged its authority. The Church was thus in favor of an interpretation of Valtorta’s work as literature and fiction, but not as a revealed text.

Even though the *Evangelo* (Valtorta 1993–1998) is surely Maria Valtorta’s most famous and controversial text, my analysis instead delves into *The Book of Azariah* because this latter is an exegetic work, namely, a commentary on the passages of the Scriptures read during each mass. Indeed, my goal here is not to reconstruct the debate about Vlatorta’s work, but rather to study how the interpreter’s subjectivity and authority is built inside the text and how her interpretation is legitimated. Indeed, as the above-quoted passage from the “Osservatore Romano” mentions, the character of the narrator and interpreter seems to constitute an element running across all of Maria Valtorta’s various writings; as this character bears her name, it causes the borders between her figure, her character, and her enunciative instance as an interpreter to overlap. The *Book* is particularly interesting from the perspective adopted here, moreover, because it aims to provide guidance to people endowed with charisms, namely, spiritual gifts making them the potential receivers – and disseminators – of “private revelations.”

## 3 Mystics and political action

A further key opposition in Catholic culture is the one between action and contemplation. The lives of mystics, however, present numerous evidence that this binary opposition should instead be considered as more nuanced, as proposed in Ponzo and Galofaro (2024).9Ponzo and Galofaro (2024) present a semiotic reflection on the different forms of the saintly figures’ political engagement. In particular, mystical and hagiographic literature often represents religious or holy people who lead a life predominantly devoted to cultivating the spirit and profess an ideal of mystical or ascetic life but who, at the same time and often in spite of themselves, find themselves carrying out some kind of political action during their life. An example of this is Saint Catherine of Siena, whose writings repeatedly convey the theme of inner recollection,10Regarding Catherine’s idea of the “inner cell” and related imagery, see Ponzo (2023). of detachment from the world, but who in fact found herself having considerable political importance, for example by carrying out epistolary exchanges with the most influential figures of her time. Thus, many mystics show a willingness to abstain from public and political engagement but, despite this intention, they are nevertheless agents of political activity that sometimes, as for Catherine of Siena, ends up exerting considerable influence at the political level.

This authority enacted by the mystics on the social and political level is determined to a large extent by the fact that, by virtue of their ability to withdraw from the things of the world in a more or less radical way and instead cultivate the spirit, they represent a greater degree of closeness with supernatural interlocutors than the average believer, thereby acting as a “channel”11In the sense of Jakobson (1960). between humans and divinity. The mystic’s charismatic role is legitimated by a *knowledge* and *power* which surpass those of ordinary people. One of the fundamental practices for mystics is mediating the Scriptures. Their knowledge derives from their reading of and meditation on the sacred text, but it is often also an interpretation enlivened by experiences in which, thanks to the intervention of heavenly interlocutors, the mystics claim to reach a higher level of knowledge of the sacred text.

In other words, the political role of mystics should be read not only as direct action in the public sphere, but also in the above-discussed context of the hierarchy of interpreters of the sacred text who are entitled to speak and propose readings. The privileged perspective made possible by sacred Helpers not only makes mystics authoritative interpreters of the sacred texts, at least from the perspective of popular devotion, but also improves their understanding of the world they live in. Thanks to this understanding, they serve as points of reference in their communities, as other believers admire them for their wisdom and ask for their advice in all manner of human affairs.

## 4 Mystics’ interpretative style

Without presuming to provide an exhaustive account of the interpretative style of the mystics in the limited space of this article, I intend to highlight two partly related characteristics. The first is linked to the concept of *ruminatio*, an idea on which Massimo Leone (2015) has reflected from a semiotic perspective. Authors use this mystical and religious Latin term to designate meditation of the sacred text, a process in which the text is not only studied, thought about, and understood, but also made to enter into the depths of the being and become a constituent part of it. The second characteristic is linked to *experience*, the idea of being able to immediately relate to what is being narrated, especially in the Gospels. This idea is often connected to an ethical issue concerning the creation of direct relationship between reading and living – i.e., practicing – the Word of God, but in some cases it is connected to a more radical identification. In Ponzo (2022), I studied the testimonies concerning various mystics who report *reliving* the episodes of the Passion of Christ. The conclusion to which the research led me was that these testimonies can be considered descriptions of a coherent *interpretative style*, a very particular key to understanding the history of the Passion on an experiential basis. This *experiential* interpretative style is based on the Gospels’ story of the Passion; in this case, however, the story is interpreted, figurativized, and relived with the whole body. This experience can be conceptualized as a sort of intersemiotic translation of a story that, instead of being transposed – from verbal language to visual or audio-visual language, for example – brings into play the ecstatic consciousness and body of the mystic, thus constituting an all-encompassing experience. The audiovisual testimonies of twentieth-century mystics show that this type of experience is accompanied by a “mise-en-scène,” a mimicry in which the entire body testifies to the Passion experienced by the mystic in its various phases, in some cases clearly recognizable from the mystic’s movements.

Many mystics authored not only exegetic works but also rewritings of books or episodes of the Bible, as in the case of Maria Valtorta: all of these exegetical or narrative works are certainly the product of *ruminatio*, but at the same time they also display an experiential dimension, at least to the extent that, in most cases, the contents are presented as *mediated*, that is, as being the result not only of reflection and meditation by the mystic but also of a “private revelation” by a supernatural Helper.

## 5 Enunciative structure

The construction of the subject of enunciation in mystical works of a narrative and exegetical nature is very often based on the stratification of various levels of discourse. The result of these multiple layers is that it is not only the narrating subject who holds the authorship of and responsibility for what is being said; rather, authorship and responsibility are shared with one or more mediators, be they angels or sacred characters. The case of Maria Valtorta’s *Book of Azariah* is exemplary of this phenomenon. The commentary on the masses it contains is based on a recurring enunciation pattern in which the narrator, who expresses herself in the first person, reports that she has received a message dictated to her by her guardian angel, Azariah, and has transcribed it as direct speech. For example, here is the beginning of the Book:

11 A.M.

S. Azariah says:

“Come, let us hear the Holy Mass together. Today’s liturgy, while addressing everyone, is addressed in particular precisely to you, God’s extraordinary instruments …

Look? ‘O God whom you see … ’

Here. Humility: One of the Essential Virtues in Extraordinary Instruments…” (Valtorta 2007 [1982]: 13)

The commentary on this first mass is particularly interesting because, precisely at the beginning of the book, it brings to the forefront (albeit in an indirect way) a reflection on the status of the mystic and the relationship between her, her angelic interlocutor, and the divinity: indeed, the reflection is directed to those who have special spiritual charisms. In this frame, the narrator’s voice expresses itself as “I” while in the speech dictated by Azariah it becomes the “you” to whom the angel’s words are addressed. In this commentary, the narrator’s voice is inserted into a further and plural “you,” that is, it is identified with a category of individuals endowed with superior spiritual capacities.

Such individuals are invited to practice humility in consideration of their subordinated enunciative role. Indeed, they are admonished to remember that they are just “mouths,” not “sources.” In fact, the angel says, the existence of the river and its mouth depends on the source, so it is right to praise only the “Source.” Again, those who have special charisms are described as “instruments,” as individuals who *have received* gifts for which they must be grateful. These exhortations therefore contribute to diminishing the agency, as well as the authorship, of the mystic, instead granting to the divine author the agency involved in producing the message. Implicitly, however, they exalt the mystic’s role as a mediator between the divinity and humans, investing the message she conveys with a connotation of authenticity sanctioned by the divine interlocutor: the latter consecrates her as an “instrument” delegated by the deity to transmit their message. It is not surprising, therefore, that the second point touched upon by the comment is sincerity:

You, soul who has been entrusted to me, know how many times the Tempter seduces, proposing to make comedies, to add frills, to amaze, to appear even more! The *Great Danger! Only those who know how to resist and be what God does them, and nothing more, retain the gift and remain an instrument.* How trembling I have seen you tempted every time! And with what praise of glory have I blessed the Lord and thanked the Heavenly Court for having helped you to endure, every time I saw you come out of the trial tired, suffering, but more mature, but victorious! (Valtorta 2007 [1982]: 14)

The value advocated here is ensuring the authenticity of the message through a lack of personal interpolation on the part of the mystic. The affirmation of this principle, however, is combined with a Sanction granted by Azariah: the angel’s praise for the mystic’s ability to refrain from embellishing the contents of which she must act as the bearer further strengthens her authority as a reliable instrument of divine communication. The angel compares moreover the “extraordinary instruments” to Paul of Tarsus who, “raptured to the third heaven … heard arcane words that it is not lawful for a man to utter.” The narrator is thus compared to Paul, placed in an inferior position but invested with the same mission:

You are not caught up to the third heaven, but you hear arcane words, but they are *given to you to be given*. So you are far inferior to Paul. And yet: hear the words of the one who deserved to be raptured so high as to hear the secrets, the mysteries of God! (Valtorta 2007 [1982]: 15)

At the conclusion of the commentary on this mass, the frame closes as follows: “And my Azariah, who spoke with wonderful sweetness, greets me with a smile and is silent …” (Valtorta 2007 [1982]: 16).

In Azariah’s discourse, the mystic is placed in a macro-category of mediators between the divine word and human recipients. This macro-category is hierarchically organized in such a way that the highest position is occupied by the angels, followed by Paul (whose humanity is emphasized) and then the “extraordinary instruments,” such as the mystic who reports Azariah’s speech. The “political” problem with this categorization is immediately apparent: in the culture under consideration, Paul is considered an *inspired* author and his works are part of the biblical canon. Mystics are declared to be inferior, but the difference is only one of *degree* rather than quality. This raises the problem of the placement of the mystic’s works. While a community of interpreters partly composed of religious people and clergymen considers these words to be the fruit of a “private revelation,” the Church’s authorities have officially denied this status, recognized a qualitative difference and considered them fictional literature instead. This Sanction places the mystical writings quite distant from the central position occupied by the canon of the sacred texts, and the figure of the mystic is relegated to a marginal and conflictual position with respect to the ecclesiastical hierarchy.

## 6 The Sanction

The mystic’s discourse thus forcefully claims a positive Sanction granted by supernatural mediators who take the place of the divinity as interlocutors, a Sanction that the mystic communicates in the form of direct testimony. And yet the *Book of Azariah,* as many other texts of the same genre, also presents a further level of Sanction. This further level is a Sanction by human agents, one which tends to use the same parameters and the same categories on which the mystic’s own discourse is based. This Sanction is an almost essential filter for the fruition of the text, as it is inscribed in the paratext in the form of a preface and, as such, is designed to frame and precede the reading of the Valtortian text in a way that influences the readers’ interpretation from the beginning. I would like to point out three features of this preface, which is signed by the clergyman Fr. Corrado M. Berti O.S.M. The first feature is that it provides some very detailed information about the situation of the enunciation:

Maria Valtorta, who was ill, wrote this volume … in Viareggio, in Via Antonio Fratti, in the house that now bears the house number 257, she wrote it while lying in bed, with the notebook resting on her knees, in her own hand, with one of the fountain pens currently preserved in the archive, on the spur of the moment, without possessing or consulting suitable books, without corrections, previous schemes or revision of any kind. (Valtorta 2007 [1982]: 5)

The first part of this passage serves to place the text precisely in the time and space of the enunciation and gives us information about the empirical author. These traits can be explained as an attempt by the paratext to emphasize the text’s status as testimony. As Ricoeur (2000: 204) reminds us, what characterizes testimony is precisely an assertion of reality inseparable from the subject who testifies: we can therefore hypothesize that the reference to details about the situation and subject of the enunciation is strategic in that it reinforces this act of designating the subject as a witness. The reference to Valtorta’s infirmity and inability to consult pertinent works, on the other hand, refers to the value of “sincerity” and that of a revelation which, due to its exceptional quality, must necessarily be of a supernatural nature, thus surpassing the skills and knowledge of the “channel” that mediates and enunciates it.

The second feature concerns the introduction of the Sender who places himself between the text and the reader as an ecclesiastical authority, but also as the publisher of the text itself. In fact, the preface opens by informing the reader that Maria Valtorta had proposed the title “Angelic Masses, Directions” for her book, but that this title was not considered appropriate as it could give rise to misunderstandings on the theological level. This Sender, of which the author of the preface is an actor (but probably not the only one) therefore reports the original title but, at the same time, also communicates that he has changed it and explains his motivations for doing so, clearly giving the idea that the text as it is presented to us does remain faithful to the original but has also undergone a process of editing: while on the one hand providing indications about the situation of the enunciation can be read as a strategy of *embrayage*, therefore, mentioning the mediation carried out by the editors and publishers, although accompanied by a declaration of fidelity to the original, can instead be read as a *débrayage* and as a form of Sanctioning the text.

The third feature concerns the comment on the nature of the revelation received by Maria Valtorta:

Maria Valtorta … presents this writing of hers as dictated to her by an Angel, by her guardian angel, Azariah. What are we to make of such a statement? Undoubtedly, it is not impossible for an Angel, appearing or not appearing in human form or the like, to speak, say, or otherwise manifest his thoughts: the Bible itself … is teeming with angelic interventions, both for teaching and for directing. And it is incomprehensible why such phenomena cannot or should not occur again in the Church today, which is identical to that of all previous centuries. However, given the sublimity, originality, exactness, clarity of so many teachings and advice contained in this volume, if an Angel did not dictate it, undoubtedly an Angel enlightened the sick writer … Our mission is to critically publish the Valtortian Writings and not to pronounce on the various explanations that are given or will be given of the phenomenon. We reserve canonical judgment to the competent Ecclesiastical Authority alone; the strictly scientific judgment to the experts in the individual branches of knowledge. (Valtorta 2007 [1982]: 6–7)

The publisher therefore chooses not to make any claims about the nature of the revelation received by Maria Valtorta; rather, he bows to a superior authority, namely, the ecclesiastical one. Although he admits the possibility of angelic intervention, the publisher seems perplexed about the concept of “dictation,” proposing instead a more generic idea of inspiration defined as “enlightenment.” To understand this cautious stance, it is necessary to take into account that, as mentioned above, the monumental rewriting of the Gospel by Maria Valtorta – based on the same enunciative strategy as *The Book of Azariah* – was firmly condemned by the Church. Over the course of time, the ecclesiastical authorities have always remained very skeptical about the authenticity of Valtorta’s revelations. If we look at the critique of the rewriting of the Gospel published anonymously in “L’Osservatore Romano” in 1960, it is evident that the judgment has to do not only with theological motivations but also, in part, with a gender bias:

according to the author (or rather female author) of this work, the Blessed Virgin has the facetiousness of a modern propagandist, she is always present everywhere, she is always ready to give lessons in Marian theology that are updated in reference to the very latest studies by current specialists in the field. The narrative unfolds slowly, in an almost gossipy way; here we find new facts, new parables, new characters and many, many women following Jesus. Some pages, then, are rather scabrous and are reminiscent of certain descriptions and scenes in modern novels, such as, to cite just a few examples, the confession made to Mary by a certain Aglae, a woman of ill repute (vol. I, p. 790 ff.), and the not very edifying account on p. 887 ff. of vol. I, [of] a dance performed, certainly not demurely, before Pilate in the Praetorium (vol. IV, p. 75), etc. At this point a particular observation arises spontaneously: the Work, by its nature and in accordance with the author’s and Editor’s intentions, could easily make its way into the hands of the nuns and the students at their colleges. In this case, the reading of such passages as those mentioned above could hardly be undertaken without spiritual danger or harm.^7^

Resistance to women writers on the part of patriarchal society, widely investigated even outside of the religious sphere, may be a factor that helps us to understand the complex enunciational structure enacted in mystical texts providing interpretations of the Scriptures, especially when mystics are women who have to legitimate their role not only as readers, but as *disseminators of an interpretation* of the sacred texts. In fact, the female interpreter renounces her own agency in the interpretative process itself and delegates it instead to a supernatural Helper, reserving for herself the role of “extraordinary instrument” called on to communicate the Other’s words. On one hand, this kind of interpretation empowers the human medium as a privileged interlocutor engaging with angels and the sacred in general. On the other hand, however, it disempowers the woman-interpreter, depriving her of an intellectual role in the interpretation. Moreover, by claiming a supernatural origin, this kind of discourse places her outside any possibility of a hermeneutical exchange, human dialogue or debate between peers, thus making of her an isolated figure and excluding her from the interpretative conflicts animating the more central regions of the semiosphere. Her only interaction is being subjected to an either-or Sanction on the part of the ecclesiastical authorities: they can either endorse or refuse the authenticity of the revelation she has received, and thus endorse or refuse the interpretation she proposes *in toto*.

## 7 Conclusions

**Figure 1:**
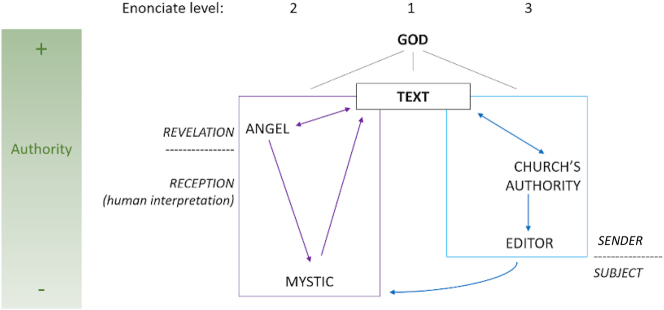
Enunciative scheme.

The case study discussed herein shows that the texts with which mystics comment on passages of the Scripture are embedded in a complex enunciative scheme based on a relationship of metatextuality that can be represented as in Figure 1.

If the Scriptures provide the first-degree text, the interpretative discourse of mystics is placed in the second degree and the discourse that sanctions the mystical text in the third degree. The mystic is positioned in the discourse in relation to a Sender embodied by a series of hierarchically organized actors, some supernatural and others human. The former are upstream of the interpretation and enunciation, so to speak, while the latter are downstream, pronouncing an a posteriori Sanction of the interpretation. If we consider the sacred text as the primary point of reference in this case as well, we see that it is accessible to readers of the mystic’s text in an extremely mediated way. Considering Valtorta’s text from the point of view of its syntagmatic organization, we can say that the reader is first of all confronted with the preface, in which the first mediator – that is, the editor – is placed; then there is the mystic, who takes the floor in the first person only to immediately cede it to the angel, whose speech is reported in a direct form. It is only at this level of the utterance that we find direct quotations and commentaries of the sacred text.

In the exegetic work analyzed here, a great deal of energy and space are devoted to constructing, enunciating, and sanctioning the enunciative role of the mystic. As mentioned above, the fundamental features of this role are the fact that the mystic serves as a channel (that is, a vehicle for a communication that is not her own but is rather reported by her as a witness) and her sincerity, or authenticity, in reporting the message. These criteria inform both the subject’s identity construction and the recipients’ Sanction. A useful concept for problematizing both of these criteria, and revealing the enunciative strategies to which they are linked, is that of “interpretative style.” Indeed, this notion serves to highlight how interpretative processes and criteria must be considered from the perspective of specific semiotic ideologies, with the attendant implications in terms of power and authority. This political dimension sometimes comes to be even more important than the interpretation of the text for its own sake, so much so that the interpretation of the sacred text can appear mainly as part of an argumentative and persuasive strategy aimed at supporting or refuting a certain idea of religion, society, institutions, etc.

This idea of “interpretative style” also helps keep the focus on what can be called the “rhetoric of interpretation,” that is, the way that metatexts interpreting sacred texts are based on enunciative strategies, actantial patterns, and recurring figures. In the case of some mystics – especially women – interpreting sacred texts, the stratification of the subjects of enunciation can be read precisely as an apologetic strategy put in place to legitimize interpreters who represent a minority and who go on speaking despite being ostracized by those who hold power and authority over the interpretation of sacred texts. As a consequence, there is not necessarily any substantial difference in the content of the interpretations or how they make their way into the mind of the interpreter: what is most remarkable and interesting, from the perspective adopted herein, is instead the energy, time, and amount of space dedicated in these interpreters’ texts to constructing themselves as subjects and, in the paratextual and metatextual apparatuses, being (or not being) sanctioned as such: the weight borne by the construction of authorial subjectivity is a clear indication of the political problem that underlies not so much the individual interpretation in question but rather the overall interpretative style in which it is inserted, a style which is in turn subject to negotiation among various political-social-cultural forces. In general, mystics tend to attract a great deal of attention and devotion in their communities. In light of their charismatic role, their position as interpreters of the Scriptures is of the greatest importance to the Church as the institution claiming the right to control the interpretation of the sacred text and the hierarchy of its interpreters.
